# Autosomal dominant familial neurohypophyseal diabetes insipidus caused by a novel missense mutation in *AVP* gene in a large Italian kindred

**DOI:** 10.1007/s12020-021-02830-x

**Published:** 2021-07-28

**Authors:** Carlotta Marzocchi, Silvia Cantara, Alfonso Sagnella, Maria Grazia Castagna, Marco Capezzone

**Affiliations:** Department of Medical, Surgical and Neurological Sciences, University of Siena, Siena, Italy

**Keywords:** Familial neurohypophysial diabetes insipidus, AVP gene, Novel mutation, AVP-NPII protein, p.C52S

## Abstract

**Purpose:**

Familial neurohypophysial diabetes insipidus (FNDI), commonly caused by autosomal dominant *arginine vasopressin (AVP)* mutations, is a rare condition in which vasopressin fails in regulating body’s level of water with final polyuria and polydipsia. Genetic testing in familial cases of FNDI should be carry out to ensure adequate treatments and avoid disease manifestations especially in infants.

**Methods:**

In this study, we investigated three-generations of a large Italian family with clinical diagnosis of familial central diabetes insipidus for the presence of potential pathogenic mutations in the AVP gene.

**Results:**

We identified a heterozygous missense mutation (c.154 T > A; p.C52S) in AVP gene in all affected members studied of a large Italian family. In *silico* tools were used to investigate the pathogenic role of the mutation and three-dimensional protein structure predicted that the p.C52S impairs disulfide bridges formation resulting in misfolding of the protein.

**Conclusions:**

This is the first study that identified a novel missense p.C52S mutation as causative of central diabetes insipidus in a large Italian pedigree.

## Introduction

Familial neurohypophysial diabetes insipidus (FNDI) [OMIM#125700], an autosomal dominant disorder, comes in many forms that are differentiated by the inheritance pattern and the underlying genetic lesion. The disease is caused by mutations in the vasopressin-neurophysin 2-copeptin protein (AVP-NPII), in wolframin (WFS1) or in proprotein convertase subtilisin/kexin type 1 (PCSK1) genes [1]. Clinically, FNDI is characterized by polyuria (>50 mL/kg), compensatory polydipsia and increased thirst (water intake of up to 20 L/day) and failure to concentrate urine. Usually symptoms manifest at an age of several months to several years (typically between 1 and 6 years of age), with a gradual onset due to the progressive destruction of vasopressinergic neurons [2, 3]. The severity of polyuria may vary considerably between different families and even among siblings carrying the same mutation. Desmopressin (DDAVP) administration represented the standard treatment. The frequency of FNDI is currently unknown, although autosomal dominant forms account approximately between 3.5 and 8%, with a similar prevalence among males and females, in series of patients with central diabetes insipidus (DI) [4, 5].

The *AVP* gene, located on the short arm of chromosome 20 (20p13), consists of three exons and encodes for the prepro-vasopressin precursor (prepro-AVP) that consists of the 19-amino acid signal peptide (exon 1), the 9-amino acid arginine vasopressin (AVP) peptide (exon 1), the 93-amino acid carrier protein neurophysin 2 (NPII) (exon 1, 2, 3) and the 39-amino acid copeptin (exon 3) (https://www.uniprot.org/uniprot/P01185) [6]. AVP is a posterior pituitary hormone, produced in the supraoptic nucleus and paraventricular nucleus of the hypothalamus. It plays a pivotal role in water balance by promoting reabsorption of free water through the V2 receptor in kidney and its synthesis and secretion is regulated by plasma osmolality (or serum Na) in physiological conditions [7]. When AVP-NPII is inadequately produced or it is not perfectly functioning, a condition called DI occurs. Most autosomal dominantly inherited cases of neurohypophyseal diabetes insipidus are due to AVP mutations located in the NPII moiety or in the signal peptide whereas few mutations are located directly in the AVP moiety [8]. Currently, 87 disease-causing mutations in *AVP* gene have been reported in The Human Gene Mutation Database (HGMD) including mainly missense mutations and rarer deletions, small indels and splice site mutations [9].

In this study we investigated three-generations of a large Italian family with clinical diagnosis of FNDI for the presence of potential pathogenic mutations in the *AVP* gene.

## Patients and methods

### Patients

**Fig. 1:**
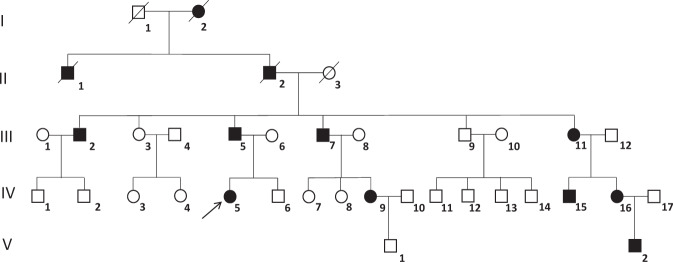
Pedigree of the family objective of the study. Generation and subject numbers are indicated. Affected members are indicated in black. Proband is pointed by an arrow. /=deceased, ○=females, ϒ=males

Nineteen/31 (61.3%) members belonging to three-generation of a large FNDI kindred were studied. The pedigree (Fig. 1) is consistent with an autosomal dominant mode of transmission. Nine/19 (47.4%) members (III:2; III:5; III:7; III:11; IV:5; IV:9; IV:14; IV:15; V:2) had polyuria and polydipsia since early childhood (since 5–10 years) and received treatment with desmopressin (DDAVP). The proband (patient IV:5) is a 4-year-old girl who presented with polyuria and polydipsia since infancy. The patient underwent a water deprivation test, which lasted 4 h and terminated when 25 mcg of DDAVP was administered intranasally that confirmed the diagnosis of central diabetes insipidus. The MRI of the pituitary did not reveal pathological images. Similar results were reported in the other FNDI members. All patients are in excellent health and doing well on treatment with DDAVP.

All procedures performed in this study were in accordance with the local ethical committee (Comitato Etico Regione Toscana, Area Vasta Sud Est, AOUS).

Informed consent has been required and signed by each patients enrolled in the study.

### PCR amplification of *AVP* gene

Genomic DNA was extracted from peripheral blood leukocytes with QIAamp^®^ DNA blood mini kit (Qiagen) according to manufacturer’s instruction and concentration was assessed with Nanodrop One (Thermo Scientific). All three coding exons of the *AVP* gene (Ensembl gene ID: ENSG00000101200, transcript ID: ENST00000380293.3, NM_000490.5) were analyzed. For each patient, 100 ng of DNA were amplified by PCR using specific primers pairs: exon 1 forward 5′- GCGGACATAAATAGGCAGCC-3′ and reverse 5′-CCACCCATGACTTCCCTCTT-3′; exon 2 forward 5′-GTTCCCCTCCAACCCCTC-3′ and reverse 5′-CGGGAGGACGTGTGAGCA-3′; exon 3 forward 5′- GTTTGCTGCAACGACGGT -3′ and reverse 5′- ACCTCTCTCCCTTTCCCTCT-3′. PCR reaction was performed with Fast Start Taq DNA Polymerase (Roche) with MgCl_2_ at a final concentration of 1.5 mM. Annealing temperatures were 60 °C for exon 1 and 3 and 55 °C for exon 2. PCR products were run on 2.5% agarose gel and purified with GenElute PCR Clean-Up Kit (Sigma-Aldrich).

### DNA sequencing

Sequencing was performed using the BigDye Terminator v1.1 Cycle Sequencing Kit (Applied Biosystems) and BigDye Xterminator Purification Kit on automated DNA capillary sequencer (Applied Biosystems 3130xl Genetic Analyzer).

### Bioinformatics analysis

PredictSNP (https://loschmidt.chemi.muni.cz/predictsnp/) software was used to predict the effect of the mutation in the amino acid sequence of AVP-NPII. Swiss-Model and PyMOL software were utilized to build the three-dimensional protein structure of wild-type and mutant AVP-NPII protein. SWISS-MODEL Repository 3D protein structure model P01185 (PDB ID: 7kh0.1) Homo sapiens (Human) was used to predict the effect of mutation.

## Results

### Identification of the p.C52S mutation in *AVP* gene

**Fig. 2:**
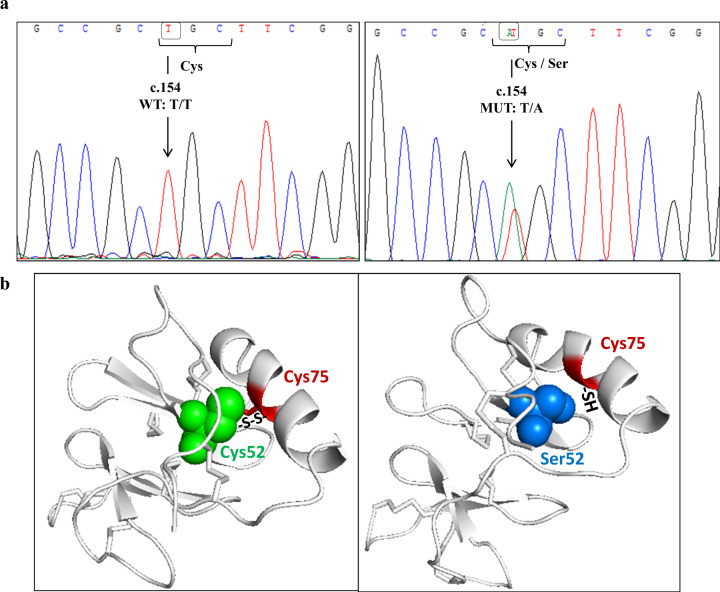
**a** *AVP* gene’s exon 2 chromatogram for two patients. On the left: wild-type sequence with T allele at position c.154; On the right: mutant heterozygous sequence with the mutation at position c.154 (T → A) that leads to an amino acid substitution from cysteine (Cys) to serine (Ser). **b** Three-dimensional structure of AVP-NPII protein. On the left: Wild-type protein with cysteine at position 52 (Cys52) (represented as green spheres) that forms disulfide bridge (S-S) with cysteine at position 75 (Cys75) (depicted as red region). On the right: mutant protein with serine at position 52 (Ser52) that prevents the formation of disulfide bond with the sulfhydryl groups (-SH) of cysteine at position 75 (Cys75). Ser52 and Cys75 are represented as blue spheres and red *sticks in helix* region, respectively

The entire coding region of the *AVP* gene was analyzed in 19 members of an Italian family with FNDI. From DNA sequencing, we detected a novel missense mutation caused by the substitution of a thymidine with an adenine at position 154 in exon 2 (c.154 T > A), that lead to the replacement of cysteine for serine at codon 52 (p.C52S). All affected members (47.4%) were heterozygous for the missense mutation c.154 T > A, while the others subjects were homozygous for the wild-type allele T (III:3; III:9; IV:2; IV:3; IV:4; IV:6; IV:8; IV:13; IV:14, V:1). An example of sequencing chromatogram of a normal and mutant sequence is shown in Fig. 2a. Apart this mutation, no other variant in the *AVP* gene was found in the family members.

### Prediction of the effect of the p.C52S mutation *in silico*

PredictSNP is a consensus classifier that analyzed aminoacid or nucleotide substitution(s) and gives back a score predicting possible impact on protein function employing six best performing integrated tools (MAPP, PhD-SNP, PolyPhen-1, PolyPhen-2, SIFT and SNAP). When analyzed, the p.C52S mutation is predicted to be severely deleterious with an overall score of 87% (MAPP: 77%, PhD-SNP: 88%, PolyPhen-1: 74%, PolyPhen-2: 81%, SIFT:79% and SNAP: 89%).

In addition, we examined the three-dimensional protein structure of the wild type and mutant AVP-NPII with C52S mutation (Fig. 2b) using as 3D protein structure model SWISS-MODEL Repository P01185 (PDB ID: 7kh0.1). The new missense mutation occurs in the central region of the NPII moiety at the amino acid position 52, which is involved in the formation of one of the eight intrachain disulfide bridge required for correct folding of the protein in the endoplasmic reticulum (ER). The substitution of cysteine to serine at position 52 prevents the formation of disulfide bridge with cysteine at position 75 with an impaired folding of the molecule.

## Discussion

We investigated three-generations of a large Italian family with clinical diagnosis of FNDI and treated with DDAVP for the presence of potential pathogenic mutations in the *AVP* gene and found a new substitution at residue 52 (p.C52S) in the NPII moiety of the AVP-NPII. This novel missense mutation was discovered in heterozygous conditions in nine consanguineous family members. The mutation appears to segregate in an autosomal dominant mode and it was scored pathogenic in silico. By structural analysis, the p.C52S was predicted to prevent the formation of disulfide bridge with cysteine at position 75 essential for a correct folding of the molecule in the ER. Actually, prepro-AVP is targeted to ER by its signal peptide. Here the signal peptide is removed and pro-AVP is created. Finally, glycosilation of copeptin, folding and formation of 8 disulfide bonds form a functional protein. The substitution of Cys52 by serine caused a failure in disulfide bridge formation. Cysteine (C3H7NO2S) has an isoelectric point (pl) of 5.05 and a molar mass of 121.16 g/mol. Serine (C3H7NO3) is smaller, with a pl of 5.68 and molar mass of 105.9 g/mol. Moreover, by leaving the cysteine at residue 52 free to react with other cysteine residues in the NPII moiety, the mutation could also cause abnormal disulfide bridges further impairing molecule folding. Most of the cases of FNDI with autosomal dominantly inheritance are due to AVP mutations in the NPII moiety [8]. These mutant molecules are normally transcribed but, due to altered folding, are maintained in the ER leading to accumulation of cytotoxic aggregates and progressive death of vasopressinergic neurons [10]. Moreover, mutated proteins could form heterodimers with wild type protein preventing their secretion [11]. Of note, another variant of residue 52, p.C52R, has been reported in two previous studies [12, 13]. In the first paper, the authors identified at position 52 the substitution of cysteine by arginine (p.C52R, c.154 T > C) in a three generation of a Caucasian American kindred with DI suggesting that this mutation would lead to a change in the primary structure of the prepro-AVP resulting to an altered folding in the ER [12]. In a most recent paper [13], authors reported the same heterozygous mutation (p.C52R) in two members of a Portuguese family with FNDI. In vitro studies carried out, showed that the p.C52R mutation leads to an intracellular retention of the mutated protein with consequent absence of secreted AVP [13].

Different cysteine residues in the NPII moiety, involved in the formation of disulfide bonds, have been found to be mutated in autosomal dominant FNDI at position 41 [14], 58 [15], 59 [16–18], 65 [19, 20], 92 [14, 19, 21], 98 [19, 22], 104 [23, 24], 110 [21, 25] and 116 [23, 26]. All studies concluded that these mutations disrupted normal disulfide bridges leading to destabilization of NPII in the ER.

The discovery of novel genetic abnormalities in the *AVP* lets to further expand the panel of mutations associated with FNDI allowing an early diagnosis and follow-up of patients. Despite replacement therapy with DDAVP is simple and effective, the identification of specific mutation-FNDI associated may represents a target for future drugs which can avoid protein misfolding.

To our knowledge, this is the first time that the p.C52S has been associated with central DI contributing to enlarge the panel of mutations that need to be searched for during diagnosis and to guarantee an early and adequate treatment.

## Data Availability

Data available on request from the corresponding author.
